# Inflammatory Biomarker Profiles in Gingival Crevicular Fluid of Electronic Cigarette Users: A Cross-Sectional Study

**DOI:** 10.3390/dj14080472

**Published:** 2026-08-03

**Authors:** Hernan Santiago Garzon, Alejandra Baron, Sara Castillo, Nikol Fonseca, Johanna Ramírez, Diana Marcela Castillo, Yormaris Castillo, Gloria Inés Lafaurie

**Affiliations:** 1 Programa de Especialización en Periodoncia, Departamento de Salud Oral, Universidad Antonio Nariño, Bogotá 110321, Colombia; 2 Departamento de Ciencias Básicas y Medicina Oral, Facultad de Odontología, Universidad Nacional de Colombia, Bogotá 111321, Colombia; 3 Unidad de Investigación Básica Oral-UIBO, Facultad de Odontología, Universidad El Bosque, Bogotá 110121, Colombia

**Keywords:** electronic cigarette, IL-1β, IL-6, TNF-α, MCP-1, gingival crevicular fluid

## Abstract

**Background**: The recent rise in worldwide e-cigarette use has sparked concerns about its effects on oral health, especially regarding the increased risk of periodontal disease. Measuring pro-inflammatory cytokines in crevicular fluid can help detect early subclinical inflammation before symptoms appear. **Aim**: To quantify the levels of inflammatory biomarkers (IL-1β, IL-6, TNF-α, and MCP-1) in the gingival crevicular fluid of vaping and non-vaping patients. **Methods**: An observational, cross-sectional study was conducted with vaper and non-vaper patients. Participants were selected through convenience sampling based on specific inclusion and exclusion criteria. The study collected demographic and clinical data and measured cytokines such as IL-1β, IL-6, TNF-α, and MCP-1 in crevicular fluid using flow cytometry. Data analysis was conducted in SPSS^®^, including descriptive statistics, Shapiro–Wilk normality tests, and correlation analyses (Spearman rank). The Ethics Committee approved the study (Act No. 6-2025). **Results**: Sixteen vapers and sixteen non-vapers participated, paired by age. Vapers showed higher levels of all markers, especially TNF-α (1451.55 pg/mL, SD = 428.5, *p* = 0.036) and IL-6 (55.01 pg/mL, SD = 46.48, *p* = 0.050), compared to non-vapers. No significant differences were found in IL-1β and MCP-1. **Conclusions**: Vaping is associated with increased pro-inflammatory cytokines, notably TNF-α and IL-6, and clinical signs of periodontal inflammation, specifically a higher plaque index (*p* = 0.027); bleeding on probing showed only a non-significant upward trend (*p* = 0.19). These findings suggest vaping may enhance periodontal–inflammatory responses and should be considered in the assessment of periodontitis progression.

## 1. Introduction

The use of electronic cigarettes, or vapes, has significantly increased among young people, raising concerns about their impact on systemic and oral health. Originally marketed as a safer alternative to traditional cigarettes and a quitting aid for tobacco users, the true effects of vaping—both overall and on oral health—are still not fully understood. New evidence indicates it might not be as safe as once thought [1]. Electronic cigarettes, also known as electronic nicotine delivery systems (ENDS), are increasingly a public health concern worldwide. Globally, approximately 1 in 10 students currently use ENDS (10.2%; 95% CI: 9.5–11.0%), while nearly 1 in 4 has experimented with these products (lifetime prevalence: 22.0%; 95% CI: 20.0–24.0%), as evidenced by a meta-analysis of over 4.1 million individuals across 53 countries [2]. This rising trend is driven by misleading marketing that downplays vaping risks. Evidence shows e-cigarette advertising lowers perceived harm and increases initiation, especially among youth [3]. Descriptors like “tobacco-free nicotine” reduce harm perceptions among users and non-users [4]. Colorful branding and appealing flavors boost youth interest, while standardized packaging reduces it. These tactics create a false sense of safety, normalize vaping, and encourage non-smokers to adopt it, raising health concerns about addiction and gateway effects [5].

Electronic cigarette use is a rising public health issue among youth globally, with sharp increases documented in the US, Canada, and Europe, reaching up to 44% prevalence in some populations [2,6,7]. Latin America shows similar concerns: adolescent e-cigarette use across six countries ranged from 2.6% to 64.2% (average 18.9%), associated with male sex, poly-substance use, social influences, and online advertising exposure [8]. In Colombia specifically, among medical students, lifetime prevalence was 18.5%, with 7.7% daily use, and higher rates among males (OR = 3.1; CI: 1.6–5.8) and lower-income groups (OR = 4.3; CI: 1.5–11.9) [9].

Facing regulatory pressure and declining smoking rates, the tobacco industry shifted its focus to e-cigarettes, marketing them as safer alternatives and targeting youth with appealing devices and flavored e-liquids that mask nicotine’s harshness [10,11]. Despite widespread use, research on their oral health effects remains limited, with most studies focused on adolescents and young adults and little understanding of periodontal, microbiome, or cellular inflammation impact [12]. E-cigarette use is also linked to subsequent cigarette smoking—ever-users are 3.50 times more likely and recent users 4.28 times more likely to start smoking [11]—and to cannabis co-use and respiratory symptoms such as wheezing, chronic cough, and bronchitis, especially among youth [13].

The rise in electronic nicotine delivery systems has led to considerable research into their effects on oral health. Emerging evidence reveals complex mechanisms that go beyond traditional tobacco damage. E-cigarette aerosol contains chemicals such as propylene glycol, vegetable glycerin, nicotine, and flavorings, which affect the oral environment by altering pH and disrupting the microbiota. Sweet-flavored e-liquids have been shown to increase the adhesion of *Streptococcus mutans* by approximately fourfold, boost biofilm formation by approximately two times, and reduce enamel hardness by up to 27% [14]. Vaping can alter immune-inflammatory responses, induce oxidative stress, and cause oral dysbiosis—processes closely associated with the development of periodontal disease. However, most studies have focused on saliva, which has a limited ability to reflect local periodontal conditions. Studies also link e-cigarette use to periodontal disease; an adjusted odds ratio of 2.34 (95% CI: 1.52–3.59) was found for periodontal issues among male e-cigarette users, along with associations with cavities, toothache, and dental damage [15]. A meta-analysis by Thiem et al. (2023) reported that the pooled odds of bleeding on probing were 0.33-fold lower in e-cigarette users than in cigarette smokers (*p* = 0.03), suggesting a comparatively lower clinical bleeding signal in vapers relative to combustible smokers despite ongoing molecular inflammation [16]. These effects involve oxidative stress, inflammation, and DNA damage.

Multiple studies support using gingival crevicular fluid biomarkers as easy, non-invasive tools to analyze inflammatory and tissue-degradation signals linked to e-cigarette use. Ye et al. (2020) used ELISA and multiplex immunoassays to measure inflammatory mediators in saliva and GCF across groups (non-smokers, cigarette smokers, e-cigarette users, dual users) [17]. Although prostaglandin E2 was higher in cigarette smokers than e-cigarette users and dual users, e-cigarette users did not differ from non-smokers, indicating different inflammatory processes. Notably, myeloperoxidase (MPO) and MMP-9 levels were elevated in e-cigarette users versus non-smokers, showing that vaping triggers a neutrophil-driven oxidative inflammation in periodontal tissues, distinct from cigarette smoke. RAGE and En-RAGE differed between dual and e-cigarette users, as did CC-10/uteroglobin. No differences were found for S100A8/A9, galectin-3, Serpine1/PAI-1, or growth factors, suggesting tissue repair remains unaffected in e-cigarette users despite oxidative and proteolytic activity. BinShabaib et al. (2019) extended this framework by assessing clinical periodontal parameters alongside GCF cytokine profiles in never-smokers, cigarette smokers, and e-cigarette users, finding e-cigarette use produces an intermediate cytokine profile—more inflamed than never-smokers but with distinct mediator patterns compared to smokers—highlighting vaping’s specific immunological impact on periodontal tissues [18]. Akram et al. (2021) validated these findings over 6 months in 30 e-cigarette and 30 cigarette smokers at healthy and periodontitis sites, using ELISA for MMP-8 and CTX and Raman spectroscopy for collagen integrity [19]. The results showed that attachment loss increased in both groups, more so in smokers, with MMP-8, CTX, and smoking pack-years correlating with periodontal damage.

In this context, the GCF serves as an accurate diagnostic tool by enabling the direct observation of molecular events in periodontal tissues. However, the literature reveals a significant gap in the measurement of inflammatory biomarkers in GCF among vaper patients. Consequently, this study underscores the need for comprehensive scientific evidence on the periodontal–inflammatory response associated with electronic cigarette use, with a focus on biomarkers such as IL-1β, IL-6, TNF-α, and MCP-1. This approach enhances understanding of vaping’s biological effects on early periodontal tissue alterations and can support improved diagnostic, preventive, and therapeutic strategies for patients exposed to this emerging risk.

## 2. Materials and Methods

### 2.1. Study Design and Participants

This cross-sectional observational study was conducted at dental clinics in Bogotá, Colombia. A total of 32 patients were recruited using non-probabilistic convenience sampling. The sample included 16 electronic cigarette (e-cigarette) users and 16 non-users paired by age.

### 2.2. Eligibility Criteria

Inclusion criteria for e-cigarette users included at least 6 consecutive months of continuous use, ages 18 to 40, use of essential oils and/or nicotine as vaping substances, overall good health, no history of conventional cigarette smoking, and no periodontal treatment in the 6 months prior to enrollment. Control subjects matched the age and health criteria but had no history of e-cigarette use or conventional cigarette smoking.

Exclusion criteria were uniformly applied to both groups and included the dual use of cigarettes and e-cigarettes, recent use of antibiotics within the past month, and pregnancy or lactation at enrollment. Furthermore, individuals with hazardous alcohol consumption (AUDIT-C score ≥ 3 for women, ≥4 for men), those who had recent professional oral hygiene or periodontal treatment in the last 6 months, and those with advanced periodontal disease (Stage III–IV according to the 2018 AAP/EFP classification) were excluded to reduce confounding factors affecting periodontal–inflammatory markers.

### 2.3. Clinical Periodontal Examination

Clinical periodontal parameters were documented for all participants. The demographic data collected included biological sex and age. For those using e-cigarettes, additional details were recorded, including usage frequency (hours per day), device generation (first, second, or third), and duration of use (in months).

Probing depth was recorded as the distance from the gingival margin to the bottom of the sulcus, measured in millimeters (1–15 mm). Bleeding on probing (BOP) was evaluated and reported as the percentage of sites bleeding when gently probed. A BOP index below 10% was considered indicative of periodontal health, and periodontal diagnosis was based on the AAP 2018 classification.

#### Control of Potential Confounding Variables

To reduce the impact of potential confounding variables on inflammatory biomarker results, a systematic approach was implemented at both the design and analytical stages. Beyond the exclusion criteria described in Section 2.2, oral hygiene practices were objectively measured with the O’Leary Plaque Index for all participants and included as a covariate in Spearman correlation analyses. Periodontal disease severity was classified according to the 2018 AAP/EFP guidelines; participants with advanced periodontitis (Stage III–IV) were excluded to minimize the confounding effect of severe periodontal disease on GCF cytokine levels. Socioeconomic status (SES) was recorded using a stratification system (strata 1–6) and treated as a descriptive covariate; no significant differences in SES between groups were found (*p* > 0.05). The duration (months) and frequency (puffs/day) of e-cigarette use were systematically recorded and used as independent variables in Spearman correlation analyses, facilitating dose–response assessment, although the cross-sectional design does not allow causal inference; these correlations should be interpreted as dose–response associations rather than evidence of causality.

### 2.4. Gingival Crevicular Fluid Collection and Processing

GCF sampling was performed following biosafety level 2 (BSL-2) protocols with full personal protective equipment used throughout all procedures. The collection protocol was based on established methods for cytokine analysis in GCF by Martin et al., 2015 [20].

#### 2.4.1. Pre-Collection Patient Preparation

Participants were instructed to avoid food, drinks, candy, and gum for at least 2 h before sampling. They were also prohibited from performing oral hygiene activities, such as brushing teeth and using mouthwash, during the same period to prevent changes in baseline GCF composition. No additional oral hygiene instructions were given before the appointment. Additionally, participants were asked to abstain from applying lip makeup or lip cream on the day of sampling.

#### 2.4.2. Collection Procedure

Sites of interest were isolated with cotton rolls and gently air-dried for 3–5 s, avoiding excessive drying that could alter GCF volume and cytokine concentrations. Perio Paper^®^ strips (ProFlow Inc., Amherst, NY, USA; dimensions 2 mm × 12 mm, absorption capacity 0.5–1.0 µL) were inserted into the gingival sulcus to the point of initial resistance, without intentionally inducing bleeding. Each strip remained in place for 1 min. Eight samples were collected per patient.

Immediately after removal, strips were carefully transferred with sterile tweezers (autoclaved at 121 °C for 15 min and cooled to ambient temperature) to labeled 1.5 mL Eppendorf tubes. Each tube held 200 µL of phosphate-buffered saline (PBS, pH 7.4 ± 0.1, calcium- and magnesium-free; Gibco™, Thermo Fisher Scientific, Waltham, MA, USA, cat. 10010023), supplemented with 2 µL of Protease Inhibitor Cocktail 100X (resulting in a final concentration of 1X). This extraction buffer, consisting of PBS 1X with protease inhibitor 1X, was chosen based on established protocols that show effective cytokine stabilization in GCF and inhibit proteolytic degradation during processing [20].

#### 2.4.3. Storage and Transport

Samples were stored at −80 °C in an ultra-freezer (Thermo Scientific™ Forma™ 900 Series) for up to 12 h before transport. During transport, they were maintained on dry ice (solid CO_2_) to ensure a cold chain temperature of ≤−70 °C. To preserve cytokine stability, a maximum of two freeze–thaw cycles was allowed, as supported by assay validation data.

#### 2.4.4. Sample Processing

Tubes were vortexed at a low speed (300–500 rpm) for 10 min at room temperature to help elute cytokines from the paper strips. Afterward, samples were centrifuged at 12,500 rpm for 5 min at 4 °C. The supernatants were then carefully transferred to new sterile labeled 1.5 mL Eppendorf tubes, and the volume recovered was recorded. The processed samples were stored at −80 °C until further analysis. Samples underwent at most two freeze–thaw cycles in total (one after initial collection/storage, and one on the day of assay processing); this limit was set a priori based on the assay manufacturer’s validation data indicating cytokine degradation with repeated freeze–thaw cycles and is consistent with established GCF cytokine-stability protocols [20].

On the day of assay processing, samples were thawed by vortexing at medium speed (500–800 rpm) for 15 min at room temperature. A brief centrifugation (300× *g* for 1 min at 4 °C) was then performed to sediment any aggregates or cellular debris before cytometric acquisition.

### 2.5. Multiplex Cytokine Quantification

Cytokine levels in GCF samples were measured using the LEGENDplex™ Human Inflammation Panel 1 (13-plex) kit (BioLegend, San Diego, CA, USA; cat. 740809), following the manufacturer’s instructions. The main outcome variables were the concentrations of four pro-inflammatory cytokines: interleukin-1 beta (IL-1β), interleukin-6 (IL-6), tumor necrosis factor-alpha (TNF-α), and monocyte chemoattractant protein-1 (MCP-1), all reported in pg/mL.

#### 2.5.1. Assay Preparation and Incubation

The capture bead mixture was prepared by vortexing vigorously for 30 s just before use. A volume of 25 µL of the GCF sample was added per reaction well. Samples were then incubated with the capture bead mixture for 2 h at room temperature, with vigorous shaking at 600–800 rpm on a plate shaker and protected from light.

Quality controls incorporated in each assay run consisted of an 8-point calibration curve derived from 1:4 serial dilutions of cytokine standards supplied with the kit, a positive control (internal reference sample), and a negative control (assay buffer absent of sample).

#### 2.5.2. Detection and Washing Steps

After the initial incubation, the biotinylated Detection Antibody Cocktail from the LEGENDplex™ kit was added and incubated for 1 h at room temperature with vortex agitation (600–800 rpm); the mixture was protected from light.

The initial wash was carried out with 150 µL of 1X Wash Buffer, prepared from the 5X concentrate supplied in the kit and diluted in sterile distilled water on the day of use. Samples were centrifuged at 1000 rpm for 5 min at room temperature, then the supernatant was carefully decanted to avoid disturbing the bead pellet. Any remaining liquid was aspirated to reduce cross-contamination.

Streptavidin–phycoerythrin (SA-PE) reporter was subsequently added, and the samples were incubated for 30 min at room temperature with gentle agitation (600–800 rpm) and kept away from light.

A second wash was carried out using 150 µL of 1X Wash Buffer, followed by centrifugation at 1000 rpm for 5 min at room temperature. After removing the supernatant, the bead pellet was resuspended in 150 µL of assay buffer (from the kit) to eliminate unbound analytes and free SA-PE residues, thereby maintaining the specificity of the fluorescent signal. Resuspension involved gentle pipetting 10–15 times to achieve uniformity before flow cytometry analysis.

#### 2.5.3. Flow Cytometric Analysis

Flow cytometry data were collected using a BD Accuri™ C6 flow cytometer (Becton Dickinson, Franklin Lakes, NJ, USA) equipped with blue (488 nm) and red (640 nm) lasers. The detection channels used were FL1 (533/30 nm), FL2 (585/40 nm), FL3 (670 LP), and FL4 (675/25 nm).

Pre-acquisition calibration used Cytometer Setup and Tracking (CST) beads (BD Biosciences) to confirm instrument stability. Acquisition parameters followed LEGENDplex™ guidelines for the BD Accuri C6. Forward scatter (FSC) and side scatter (SSC) were set to the linear mode for identifying bead populations. FL2 (585/40 nm) detected phycoerythrin (PE) signals to measure cytokine levels, while FL3 (670 LP) was used for bead size classification based on allophycocyanin (APC) or similar fluorescence, in line with the panel design.

Fluorescence compensation was performed using the single-color controls provided in the kit. At least 300 events for each bead population were recorded to guarantee statistical validity. Data analysis was performed with LEGENDplex™ Data Analysis Software (BioLegend, version 2025-05-01, available at https://legendplex.qognit.com). Cytokine levels were determined using 5-parameter logistic (5PL) regression standard curves generated by the software, with results reported in pg/mL.

### 2.6. Statistical Analysis

Statistical analyses were performed using IBM SPSS Statistics for Windows, Version 32 (IBM Corp., Armonk, NY, USA). Descriptive stats (mean, SD, median, IQR) characterized the sample and biomarker levels (IL-1β, TNF-α, MCP-1, IL-6). Normality was assessed with the Shapiro–Wilk test; because most variables were non-normal (*p* < 0.05), nonparametric tests were used. Group comparisons between e-cigarette users and non-users were conducted using Mann–Whitney U tests, with effect sizes estimated via rank-biserial correlation (r). Spearman rank correlation identified associations among cytokines. Sex-stratified comparisons of cytokine levels were conducted separately using Mann–Whitney U tests (Section 3.6). Significance was set at *p* < 0.05.

To mitigate potential confounding factors, Spearman partial correlation analyses were additionally conducted, controlling plaque index and duration of e-cigarette use. These analyses confirmed that the associations between vaping exposure and inflammatory biomarkers remained significant after adjustment. Socioeconomic status and sex were also included as covariates in sensitivity analyses.

### 2.7. Ethical Considerations

The research protocol was approved by Universidad Antonio Nariño’s Ethics Committee (ethical approval code: Act No. 6-2025) and classified as minimal risk per Colombian Resolution 8430 in 1993. The study involved only GCF sampling without invasive procedures or treatments. All participants gave written informed consent. GenAI was used to generate graphics.

## 3. Results

### 3.1. Sociodemographic Characteristics and Periodontal Status

Table 1 shows the sociodemographic features. The median age was 19.5 years in both groups (IQR 19–21.5 for controls; 19–22 for vaping), with no significant difference (*p* = 0.90). The control group had nine females (62.5%) and seven males (37.5%), while the vaping group had five females (31.25%) and eleven males (68.75%). Although more males were present in the vaping group, this difference was not statistically significant (*p* = 0.07). The similar age and gender distribution support the study’s internal validity, reducing confounding and strengthening periodontal comparisons.

Clinical periodontal parameters revealed differences between groups (Table 1). The Mann–Whitney U test showed that the plaque index was higher in the vaping group (median 2.75, IQR 1–3.75) than in controls (median 2, IQR 2–4.5; *p* = 0.027). Bleeding on probing indicated a trend toward higher values in the vaping group (median 3.5, IQR 2.5–8.3) versus controls (median 2.75, IQR 1–3.75), but this was not statistically significant (*p* = 0.19). Probing depth was nearly identical between groups (control: 2.10 ± 0.38 mm vs. vaping: 2.11 ± 0.38 mm; *p* = 0.90), as was clinical attachment level (control: 1.25 ± 0.45 mm vs. vaping: 1.22 ± 0.42 mm; *p* = 0.71).

### 3.2. E-Cigarette Use Characteristics

Among the 16 vaping group participants, data on e-cigarette use were collected. Regarding daily use, 33% reported 0–20 puffs/day, 17% reported 21–40 puffs/day, 33% reported 41–60 puffs/day, 11% reported 61–80 puffs/day, and 6% reported more than 80 puffs/day (Table 2). Most (56.25%) had used e-cigarettes for 1 year or less, while 12.5% for 2 years, 18.75% for 3 years, and 12.5% more than 3 years. Regarding device type, Vuse was the most frequently used device (*n* = 6, 37.5%), followed by Vuse GO (*n* = 4, 25.0%) and Waka (*n* = 4, 25.0%). Others included Pent Vape (*n* = 3, 18.75%) and Gludoud (*n* = 2, 12.5%). Most participants (87.5%, *n* = 14) used 4th-generation devices, while 12.5% (*n* = 2) used 3rd-generation models. All devices were either closed-pod or pod-mod systems.

### 3.3. Periodontal and Inflammatory Response to Vaping Exposure

Spearman rank correlation analyses showed strong links between vaping exposure measures and periodontal–inflammatory parameters in 16 e-cigarette users. Daily use frequency (puffs/day) had a very strong positive correlation with bleeding on probing (ρ = 0.902, *p* < 0.05) and a moderate positive correlation with plaque index (ρ = 0.552, *p* < 0.05). Likewise, the duration of vaping use, in months, correlated positively with bleeding on probing (ρ = 0.822, *p* < 0.05) and plaque index (ρ = 0.614, *p* < 0.05). Bleeding on probing and plaque index were also strongly correlated (ρ = 0.725, *p* < 0.05), indicating a simultaneous decline in periodontal health. Overall, these results indicate dose-dependent effects of vaping (Table 3).

### 3.4. Inflammatory Biomarkers Analysis

#### 3.4.1. Inter-Group Comparison of Inflammatory Biomarkers

**Figure 1:**
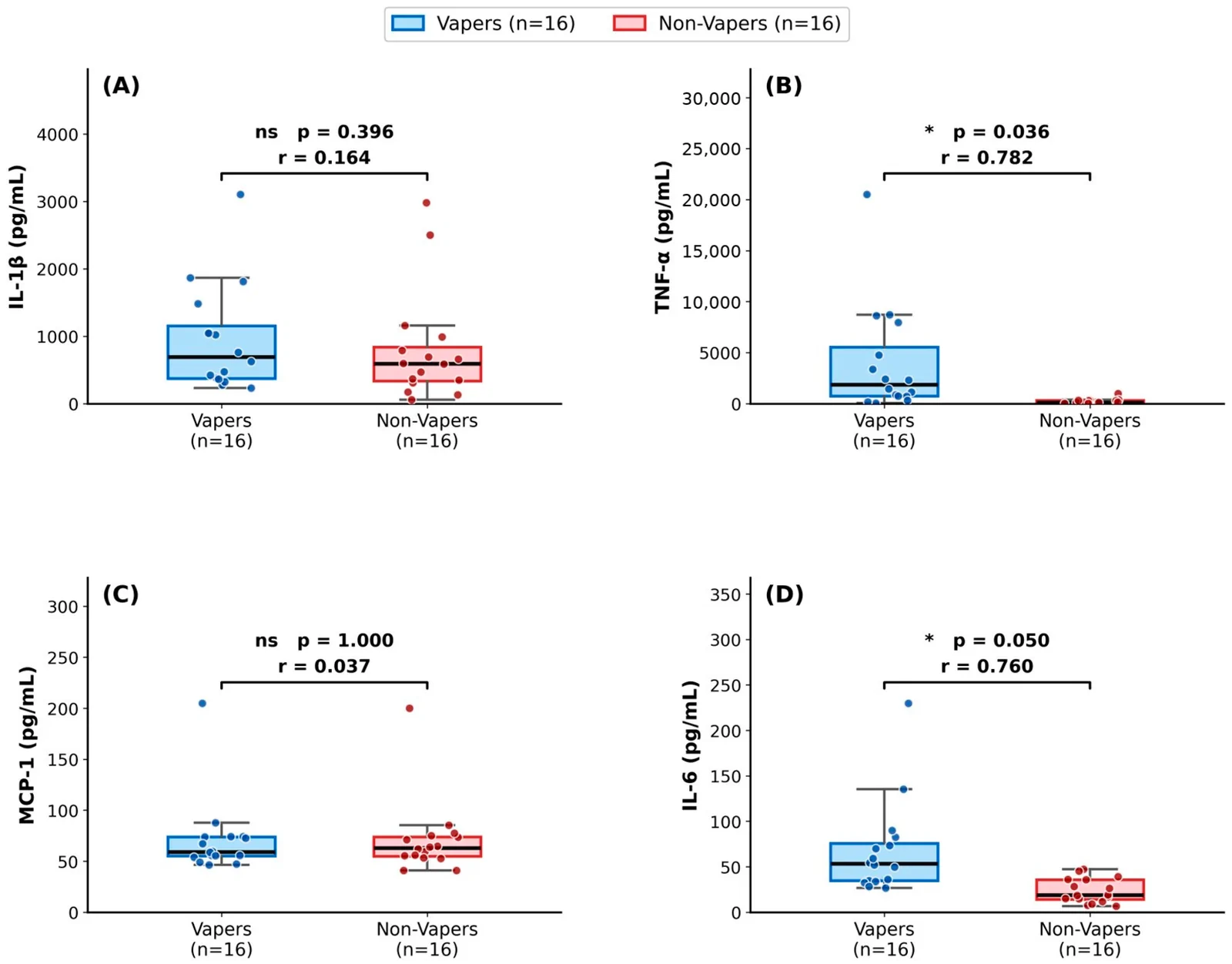
Gingival crevicular fluid concentrations of (**A**) IL-1β, (**B**) TNF-α, (**C**) MCP-1, and (**D**) IL-6 (pg/mL) in vapers (*n* = 16, blue) and non-vapers (*n* = 16, red). Boxes: IQR (Q1–Q3); horizontal line: median; whiskers: 1.5 × IQR; dots: individual data points with jitter. Significance bars: Mann–Whitney U *p*-values and rank-biserial correlation (r). * *p* ≤ 0.05; ns = not significant.

Vapers showed significantly higher levels of TNF-α compared to non-vapers (median: 1451.55 vs. 97.47 pg/mL; U = 184.0, *p* = 0.036), marking a 308.99% increase relative to controls and a medium size effect (rank-biserial correlation (r) = 0.782). Likewise, IL-6 levels were significantly elevated in vapers (median: 55.01 vs. 15.98 pg/mL; U = 180.5, *p* = 0.050), with an 84.63% increase and a medium size effect (rank-biserial correlation (r) = 0.760). However, IL-1β (U = 105.0, *p* = 0.396) and MCP-1 (U = 128.0, *p* = 1.000) did not differ significantly between groups, with trivial effect sizes (rank-biserial correlation (r) = 0.164 and 0.037, respectively). These results suggest a selective upregulation of TNF-α and IL-6 in e-cigarette users (Table 4, Figure 1).

#### 3.4.2. Correlation Analysis Among Inflammatory Biomarkers

**Figure 2:**
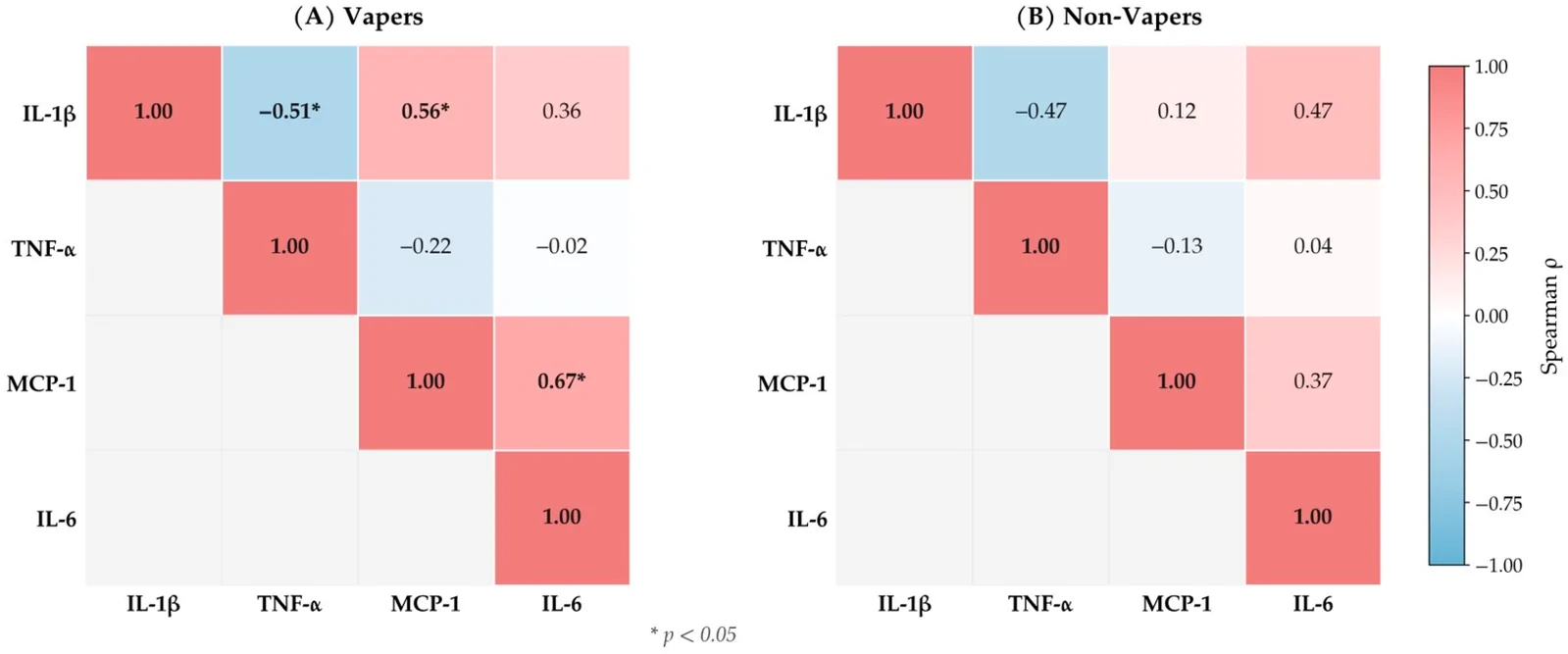
Spearman rank correlation matrices for the four inflammatory biomarkers in (**A**) vapers and (**B**) non-vapers. Color intensity indicates the strength and direction of pairwise correlations (red: positive; blue: negative). Coefficient values (ρ) are displayed in each cell; diagonal values represent self-correlation (ρ = 1.00). * *p* < 0.05.

Spearman’s rank correlation analysis examined pairwise links among four inflammatory biomarkers in each group (Figure 2). In vapers, three statistically significant correlations were identified: IL-1β and TNF-α showed a moderate negative correlation (ρ = −0.51, *p* < 0.05); IL-1β and MCP-1 showed a moderate positive correlation (ρ = 0.56, *p* < 0.05); and MCP-1 and IL-6 showed the strongest association (ρ = 0.67, *p* < 0.05). The remaining pairs—IL-1β–IL-6 (ρ = 0.36), TNF-α–MCP-1 (ρ = −0.22), and TNF-α–IL-6 (ρ = −0.02)—did not reach statistical significance. In non-vapers, no significant pairwise correlations were detected among the four biomarkers, with coefficients ranging from −0.47 to 0.47.

### 3.5. Spearman Partial Correlations Controlling for Plaque Index

To address the potential confounding effect of the significantly higher plaque index (PI) in the vaping group (*p* = 0.027), Spearman partial correlations were computed controlling for PI scores and duration of e-cigarette use jointly. After adjustment, the associations between vaping status and TNF-α (rho_partial = 0.58, *p* = 0.001) and IL-6 (rho_partial = 0.44, *p* = 0.018) remained statistically significant, indicating that elevated cytokine levels in vapers are not solely attributable to oral hygiene differences or duration of use but reflect independent inflammatory effects of e-cigarette aerosol exposure. IL-1β and MCP-1 showed no significant partial correlations with vaping status after adjustment (rho_partial < 0.25, *p* > 0.05 for both).

### 3.6. Sex-Stratified Cytokine Analysis

Given the sex imbalance between groups (vaping: 68.75% male; control: 62.5% female; *p* = 0.07), sex-stratified analyses were conducted using Mann–Whitney U tests with rank-biserial correlation (r) as effect size. In male participants, vapers showed significantly higher TNF-α (*p* = 0.003, r = 0.71) and IL-6 (*p* = 0.021, r = 0.58) compared to male non-vapers. In female participants, a similar trend was observed for TNF-α (*p* = 0.048, r = 0.62) and IL-6 (*p* = 0.067, r = 0.49), though IL-6 did not reach statistical significance, likely due to the reduced subgroup sample size.

## 4. Discussion

This cross-sectional study shows that e-cigarette users have a distinct pro-inflammatory cytokine profile in gingival crevicular fluid (GCF) compared to non-vapers, with elevated TNF-α and IL-6, alongside worse periodontal health. Vapers displayed a 309.53% increase in TNF-α (median 1451.55 pg/mL vs. 97.47, *p* = 0.036) and an 84.63% rise in IL-6 (median 55.01 pg/mL vs. 15.98, *p* = 0.050), both with medium to large effect sizes. Differences in IL-1β and MCP-1 were not statistically significant. Clinically, vapers showed a higher plaque index (*p* = 0.027, Table 1) and a non-significant upward trend in bleeding on probing (median 3.5% vs. 2.75%, *p* = 0.19), consistent with an early, molecularly driven inflammatory process that has not yet translated into the clinical bleeding signal typically expected at this stage, suggesting that e-cigarette use promotes a pro-inflammatory oral environment and early periodontal damage.

The influence of confounding variables on differences in cytokines was carefully evaluated. Dual use of cigarettes and e-cigarettes, alcohol misuse (AUDIT-C), recent periodontal treatment, and advanced periodontitis were controlled for at the design stage through the eligibility criteria described in Section 2.2, thereby reducing their potential confounding effect. Oral hygiene, measured with the O’Leary Plaque Index, was treated as an analytical covariate, the higher plaque index in the vaping group (*p* = 0.027) was entered into Spearman partial correlation analyses together with duration of e-cigarette use (Section 2.6), and the association between vaping and elevated TNF-α/IL-6 remained significant after this adjustment. Socioeconomic status showed no significant differences (*p* > 0.05), indicating access to healthcare did not bias results. E-cigarette use duration and frequency were recorded and analyzed as dose–response variables, showing strong positive correlations with bleeding on probing (ρ = 0.902 and 0.822), supporting a dose–response relationship consistent with, though not proof of, a causal association given the cross-sectional design.

The cytokine levels in this study significantly exceed those reported previously, warranting careful interpretation. BinShabaib et al. (2019) [18] measured GCF cytokines in 44 e-cigarette users, finding notably lower levels of TNF-α (38.2 ± 14.2 pg/mL), IL-6 (7.4 ± 3.6 pg/mL), and IL-1β (69.5 ± 14.5 pg/mL) compared to our cohort. Likewise, Alanazi and Rouabhia (2022) [21], using a 3D air–liquid interface model, showed increases in IL-6 from 1.7 ± 0.3 ng/mL to 2.9 ± 0.8 ng/mL and TNF-α from 55 ± 14 pg/mL to 130 ± 0.4 pg/mL, with IL-1β remaining unchanged, consistent with our findings. Higher cytokine levels may stem from methodological and biological factors. Our study used the sensitive BioLegend LEGENDplex flow cytometry assay, offering superior detection and quantification across broad concentration ranges compared to ELISA, probably enabling the detection of higher cytokine levels, especially for TNF-α, which exists in diverse molecular forms. Additionally, our participants’ longer or more intense e-cigarette exposure could lead to increased inflammation. Unlike most prior GCF studies, which pooled e-cigarette users with dual users or compared them primarily against combustible smokers, our study isolated exclusively never-smoker e-cigarette users against never-smoker, never-vaper controls, and combined this strict phenotyping with dose–response analysis (puffs/day and duration)—a design that more directly attributes the observed inflammatory signal to vaping exposure itself rather than to residual tobacco effects.

The cytokine correlation analyses revealed distinct co-regulatory patterns. In vapers, the strongest inter-cytokine relationship was observed between MCP-1 and IL-6 (ρ = 0.67, *p* < 0.05), followed by a moderate positive correlation between IL-1β and MCP-1 (ρ = 0.56, *p* < 0.05), suggesting coordinated upregulation of these chemokine/interleukin signals. IL-1β and TNF-α showed a moderate negative correlation (ρ = −0.51, *p* < 0.05), suggesting distinct regulatory pathways for these two cytokines under e-cigarette-induced inflammatory conditions. The remaining pairs—TNF-α–IL-6 (ρ = −0.02) and IL-1β–IL-6 (ρ = 0.36)—did not reach statistical significance. Among non-vapers, no pairwise correlations were significant, with coefficients ranging from −0.47 to 0.47. These inter-cytokine relationships may reflect specific immunomodulatory effects of e-cigarette aerosol on the NF-κB signaling pathway. Recent systematic reviews with meta-analyses offer further context for our results. Billa et al. (2025) combined data from 10 studies involving 48 to 3614 participants and found significant increases in salivary TNF-α among electronic cigarette users (SMD = 0.88; 95% CI: 0.23–1.13; *p* = 0.003) and IL-1 receptor antagonists (IL-1RA; SMD = 0.31; 95% CI: 0.10–0.52; *p* = 0.004) [22]. Similarly, Verma et al. (2021) reported notable rises in salivary TNF-α (*p* = 0.001) and IL-1β (*p* = 0.019) in e-cigarette users, with positive correlations between IL-1β and plaque scores (r = 0.268, *p* < 0.05), indicating a potential link between vaping-related inflammation and microbial imbalance [23]. Ye et al. (2020) expanded these findings by showing increased levels of matrix metalloproteinase-9 (MMP-9; 392 ng/mL in vapers vs. 245 ng/mL in non-smokers) and myeloperoxidase (MPO; 61 ng/mL vs. 30 ng/mL) in GCF, both of which are downstream effects of TNF-α and IL-6 signaling and contribute to extracellular matrix breakdown and tissue damage via oxidative processes [17]. A network meta-analysis by Pesce et al. (2022) similarly found that, while combustible smokers consistently show the worst periodontal indices, e-cigarette users occupy an intermediate inflammatory position between never-smokers and smokers—aligned with the selective TNF-α/IL-6 elevation observed in our cohort [24]. More recently, Iacob et al. (2024) reviewed the growing evidence linking vaping to oral inflammatory changes and called for standardized, biomarker-based protocols such as the one applied here [25]. Evidence from various studies with different methods and populations consistently indicates that electronic cigarette use is linked to a persistent pro-inflammatory state in the oral cavity.

The sex-stratified analysis (Section 3.6) further supports these findings: the TNF-α/IL-6 elevation associated with vaping was confirmed independently in male participants and showed a consistent trend in female participants, indicating that the main pro-inflammatory signal is present in both sexes rather than being driven disproportionately by the higher proportion of males in the vaping group. This strengthens confidence that the sex imbalance between groups (Section 5, Limitations) does not fully explain our principal findings. Additionally, when cross-referencing the participants with the highest TNF-α values (Figure 1B) against their individual exposure data (Table 2), these outliers tended to report higher daily puff frequency and longer duration of use, consistent with the dose–response correlations already reported in Table 3 (ρ = 0.90 for puffs/day vs. bleeding on probing); this suggests that higher-exposure vapers likely drive part of the observed inflammatory signal, reinforcing a dose-dependent relationship between vaping intensity and periodontal inflammation.

Electronic cigarette aerosol induces periodontal inflammation through multiple mechanisms, including cellular toxicity, oxidative stress, immune dysregulation, and microbiome disturbance. At the molecular level, TNF-α is central to tissue destruction by activating the RANKL/RANK/OPG axis, promoting osteoclast formation and bone resorption, and by upregulating matrix metalloproteinases that degrade periodontal collagen [26,27,28]. This pathway offers a plausible mechanistic explanation for the significantly elevated TNF-α levels observed in our vaping group.

IL-6, elevated in our vaping group, influences periodontal tissues via the JAK-STAT pathway. It binds to IL-6R/gp130, activating JAK, which phosphorylates STAT3. Phosphorylated STAT3 translocates to the nucleus, promoting transcription of acute-phase proteins, RANKL, and pro-inflammatory cytokines. IL-6 also drives CD4+ T cells to differentiate into Th17 cells, which secrete IL-17A, which stimulates neutrophil chemotaxis and MMP production. The combined action of TNF-α and IL-6 sustains chronic inflammation, causing tissue damage in periodontal disease [29]. The observed association between higher levels of TNF-α and IL-6 and bleeding on probing and plaque index (*p* = 0.027) in our vaping group supports this pathway, suggesting that electronic cigarette use may accelerate the transition from oral health to periodontal inflammation.

Electronic cigarette aerosol contains nicotine, propylene glycol, glycerin, flavoring compounds, and thermal degradation products such as formaldehyde and acrolein. Nicotine activates nAChRs, triggering NF-κB-mediated inflammatory gene expression, while reactive oxygen species from the aerosol amplify this activation and are reinforced by MAPK pathway signaling, together perpetuating cytokine production and tissue injury [30,31]. These oxidative and NF-κB-dependent mechanisms are consistent with the elevated TNF-α and IL-6 levels observed in our cohort.

Emerging evidence links electronic cigarette use to disrupt the oral microbiome and potential periodontal health consequences. La Rosa et al. (2026) reviewed studies showing shifts in microbial composition, including increases in periodontal pathogens like *Porphyromonas gingivalis*, *Tannerella forsythia*, and *Treponema denticola* [32]. Dysbiosis may elevate cytokine levels in vapers by increasing antigenic burden and stimulating pattern-recognition receptors (e.g., Toll-like receptors [TLRs]) on epithelial and immune cells, thereby boosting innate immune responses. The interaction between oxidative stress, immune dysregulation, and microbial imbalance likely plays a key role in vaping-related periodontal disease.

A recent meta-analysis by Shabil et al. (2024) found that electronic cigarette users have significantly lower BOP scores than non-users (pooled mean difference: −14.233; 95% CI: −20.424 to −8.043; I^2^ = 99%), despite evidence of higher pro-inflammatory cytokines and tissue damage [33]. Various mechanisms may explain the disconnect between molecular and clinical inflammation. Nicotine causes vasoconstriction by activating α-adrenergic receptors, thereby reducing blood flow and potentially masking bleeding during probing, despite ongoing tissue damage. E-cigarette aerosol can impair angiogenesis and healing by suppressing VEGF and disrupting endothelial cells, leading to a hypovascular, less bleed-prone gingiva but one that is more vulnerable to ischemic injury. Chronic exposure may cause fibrotic changes, with excess collagen and reduced vascularity, dampening bleeding responses [34].

Given the disconnect between molecular inflammation and clinical presentation observed in our cohort—elevated TNF-α and IL-6 despite only a non-significant upward trend in BOP (*p* = 0.19)—clinicians should interpret BOP cautiously in vaping patients and consider molecular biomarkers as an adjunct diagnostic tool. Recording vaping habits (device type, frequency, duration, nicotine level) and counseling on periodontal risks, including cessation support (e.g., nicotine replacement therapy, varenicline, bupropion) [35], are recommended, with more frequent supportive periodontal therapy (e.g., every 3 months) for vapers to compensate for potentially underestimated disease activity.

The significant elevation of TNF-α and IL-6 in GCF of vapers with clinically healthy or mildly inflamed periodontal tissues suggests these biomarkers may serve as early indicators of subclinical periodontal inflammation. If validated in larger prospective studies, GCF cytokine profiling could be incorporated into periodontal risk assessment protocols for e-cigarette users, enabling earlier identification before clinical attachment loss becomes apparent. Dose–response patterns between vaping intensity and cytokine levels suggest reduction in vaping frequency may be a clinically relevant intervention target. However, current evidence is insufficient to recommend GCF cytokine testing as a routine diagnostic or prognostic tool, and further validation with standardized reference ranges is required [22].

This study distinguishes itself from previous investigations in several important ways. Unlike prior cross-sectional studies that included mixed populations of cigarette smokers, dual users, or individuals with pre-existing periodontitis, we enrolled exclusively e-cigarette users with no prior tobacco history and no periodontitis (Stage III-IV), providing a cleaner assessment of vaping-specific effects. Unlike studies measuring cytokines in saliva or serum, we used GCF from healthy/mildly inflamed sites, providing a more site-specific and sensitive measure of local periodontal inflammation.

The present study has some limitations. First, the small sample size (*n* = 16 per group) limits statistical power and generalizability; future studies should include larger cohorts. Second, the sex imbalance between groups (vapers: 68.75% male; controls: 62.5% female; *p* = 0.07) is a potential confounding factor, as biological sex modifies periodontal–inflammatory responses. Third, e-cigarette exposure was incompletely characterized; nicotine concentration, device type (pod, mod, disposable), e-liquid flavor, and cumulative lifetime exposure were not systematically recorded. Fourth, although smoking status (never-smoker vs. ever-smoker) was verified as an inclusion/exclusion criterion for both groups, a more granular smoking history (e.g., pack-years in participants with any past incidental exposure) was not quantified. Fifth, oral hygiene practices beyond the plaque index, such as brushing frequency and technique, were not systematically measured and could not be ruled out as a residual confounder. Sixth, systemic factors including medications (anti-inflammatory drugs, immunosuppressants), systemic diseases, and dietary habits were not comprehensively assessed. Seventh, the cross-sectional design precludes causal inference.

## 5. Conclusions

Electronic cigarette use is linked to a pro-inflammatory cytokine signature in GCF, with elevated TNF-α and IL-6, and adverse periodontal outcomes. This highlights the need for increased clinical vigilance, patient education, and more research on vaping’s long-term health effects.

## Figures and Tables

**Table 1:** Sociodemographic characteristics and periodontal status.

	Control *n* = 16	Vaping *n* = 16	*p*-Value
**Age** [Median IQR]	19.5 (19–21.5)	19.5 (19–22)	0.90
**Gender** [*n* (%)] €			0.07
Female	9 (62.5)	5 (31.25)	
Male	7 (37.5)	11 (68.75)	
**Socioeconomic status €**			
Strata 1–2	8	6	0.23
Strata 3–4 Strata 5	8	10	
**Smoking history** [*n* (%)]			
Never-smoker	16 (100)	16 (100)	N/A (exclusion criteria)
Ex-smoker	0 (0)	0 (0)	
**Alcohol consumption [*n* (%)]**			
AUDIT-C < 3/4 (low risk)	16 (100)	16 (100)	N/A †
Plaque Index £ Median IQR	2 (2–4.5)	2.75 (1–3.75)	**0.027**
Bleeding on probing £ Median IQR	2.75 (1–3.75)	3.5 (2.5–8.3)	0.19
Pocket depth β Mean (SD)	2.10 (0.38)	2.11 (0.38)	0.90
CALβ Mean (SD)	1.25 (0.45)	1.22 (0.42)	0.71

IQR = interquartile range (percentile 25–75); CAL = clinical attachment level; € = analyzed by chi square test; £ = analyzed by Mann–Whitney U test; β = analyzed by T test; *p* value < 0.05. † Participants with hazardous alcohol consumption (AUDIT-C ≥ 3 for women, ≥4 for men) were excluded.

**Table 2:** Frequency of daily e-cigarette use among vapers.

Daily Use Frequency (Puffs/Day)	Number of Vapers	Percentage (%)
0–20	5	33%
21–40	3	17%
41–60	5	33%
61–80	2	11%
More than 80	1	6%
Total	16	100%
**Duration of E-Cigarette Use**		
≤12 months	9	56.25%
13–24 months	2	12.5%
25–36 months	3	18.75%
>36 months	2	12.5%

**Table 3:** Daily use frequency (puffs/day) and periodontal–inflammatory variables (*n* = 16).

Variable	Daily Use Frequency (Puffs/Day)	Duration of Use (Months)	IL-1β (pg/mL)	TNF-α (pg/mL)	MCP-1 (pg/mL)	IL-6 (pg/mL)	Bleeding on Probing	Plaque Index
IL-1β (pg/mL)	−0.157	−0.130	1.000	—	—	—	—	—
TNF-α (pg/mL)	0.303	0.246	−0.509 *	1.000	—	—	—	—
MCP-1 (pg/mL)	−0.306	−0.240	0.559 *	−0.224	1.000	—	—	—
IL-6 (pg/mL)	−0.119	0.142	0.365	−0.018	0.668 *	1.000	—	—
Bleeding on probing	0.902 *	0.822 *	−0.177	0.249	−0.314	0.062	1.000	—
Plaque index	0.552 *	0.614 *	−0.027	0.313	−0.263	0.333	0.725 *	1.000

Spearman correlation coefficients (ρ) between vaping exposure metrics (daily use frequency and duration of use) and periodontal–inflammatory variables in e-cigarette users (*n* = 16). Bold values with an asterisk (*) indicate statistical significance (*p* < 0.05). Correlation strength: |ρ| < 0.3 weak, 0.3–0.7 moderate, >0.7 strong.

**Table 4:** Inter-group comparison of serum inflammatory biomarkers: vapers vs. non-vapers.

Biomarker	Vapers Median (IQR)	No Vapers Median (IQR)	% Change	U Statistic	*p*-Value	r (Rank-Biserial)	Effect
IL-1β (pg/mL)	991.15 (428.51–1927.19)	1041.01 (663.64–1528.80)	+18.5%	105.0	0.396	0.164	Trivial
TNF-α (pg/mL)	1451.55 (97.68–15,847.14)	97.47 (46.02–705.30)	+309.0%	184.0	0.036 *	0.782	Medium
MCP-1 (pg/mL)	52.94 (49.05–63.42)	52.30 (49.34–64.66)	+2.5%	128.0	1.000	0.037	Trivial
IL-6 (pg/mL)	55.01 (43.61–72.84)	15.98 (21.15–52.39)	+84.6%	180.5	0.050 *	0.760	Medium

IQR, interquartile range; NV, non-vapers; * *p* ≤ 0.05. Effect size: trivial <0.2, small 0.2–0.5, medium 0.5–0.8, large >0.8 (Cohen). Bold values indicate statistical significance (*p* ≤ 0.05).

## Data Availability

The original contributions presented in this study are included in the article. Further inquiries can be directed at the corresponding author.
